# Mediterranean diet during pregnancy and infant neurodevelopment: A prospective birth cohort study

**DOI:** 10.3389/fnut.2022.1078481

**Published:** 2023-01-16

**Authors:** Fei-cai Dai, Peng Wang, Qiong Li, Lei Zhang, Li-jun Yu, Lin Wu, Rui-xue Tao, Peng Zhu

**Affiliations:** ^1^Department of Maternal, Child and Adolescent Health, School of Public Health, Anhui Medical University, Hefei, China; ^2^MOE Key Laboratory of Population Health Across Life Cycle, Hefei, China; ^3^NHC Key Laboratory of Study on Abnormal Gametes and Reproductive Tract, Anhui Medical University, Hefei, China; ^4^Anhui Provincial Key Laboratory of Population Health and Aristogenics, Anhui Medical University, Hefei, China; ^5^Department of Gynecology and Obstetrics, Hefei First People's Hospital, Hefei, China

**Keywords:** Mediterranean diet, pregnancy, infant, neurodevelopment, birth cohort

## Abstract

**Background:**

Embryonic neural development is associated with intrauterine nutritional status. However, few cohort studies estimated the relationship between maternal dietary patterns during pregnancy and offspring's early neurodevelopment.

**Objective:**

To examine the impact of the Mediterranean diet (MD) during pregnancy on infant neurodevelopment, including the potential mediating role of cord blood metabolites.

**Methods:**

Among 1,471 mother–child pairs in a prospective birth cohort study in Hefei, China, we investigated the associations between maternal MD score [calculated based on a validated food frequency questionnaire (FFQ)] and child neurodevelopment at infancy [assessed using Ages and Stages Questionnaires, Third Edition (ASQ-3)]. The cord blood metabolic markers (including C-peptide, high-density lipoprotein-cholesterol, low-density lipoprotein-cholesterol, total cholesterol, and triglycerides) were measured.

**Results:**

The MD score was negatively associated with communication domain developmental delays in infants [relative risk (RR) with 95% CI: 0.34 (0.16, 0.72)]. Compared with girls, boys born from mothers with lower MD scores during pregnancy were inclined to the failure of the communication domain [RRs with 95% CI for boys: 0.34 (0.14, 0.84); for girls: 0.26 (0.06, 1.18)]. Mediation analysis showed that the association between the maternal MD score and failure of communication domain mediated by C-peptide was 19.4% in boys but not in girls.

**Conclusion:**

Adhering to the MD during pregnancy was associated with a decreased risk of poor neurodevelopment, possibly mediated by lower levels of cord blood C-peptide.

## Introduction

Neurological diseases have an enormous economic and emotional impact on families and society (1, 2), and identifying modifiable factors from the early stages of life could alleviate the burden (3). Environmental factors, in particular nutritional factors, in early life are considered to be vital factors of neurodevelopment (4, 5).

Most previous studies on the relationship between maternal nutrition and child development focused on individual foods or nutrients rather than dietary patterns (6). The Mediterranean diet (MD) accentuates the intake of vegetables, legumes, fruits and nuts, unrefined cereals, fish, olive oil, moderate dairy products, and low meat (7). Epidemiological studies confirmed that MD was beneficial to multiple offspring health problems (e.g., preterm delivery, fetal birth weight, early growth, and cardiometabolic risk) (8, 9). Beneficial foods in the Mediterranean diet, such as fruits and vegetable consumption during pregnancy, have been shown to support neurodevelopment in offspring (10). A randomized controlled trial (RCT) study investigated that nuts and olive oil consumption were associated with better nervous system function in humans (11). However, the available evidence on MD during pregnancy and infant neurodevelopment is still limited.

Adhering to MD during pregnancy may reduce the maternity risk of metabolic disorders (12, 13). Data have demonstrated that maternal metabolism can change neonatal metabolism (14). Metabolic markers measured in neonatal cord blood provide insights into the placental transfer of maternal nutrients (15). Moreover, evidence shows that there exists a link between metabolic markers in early life and offspring neurodevelopment (16). Thus, the relationship between MD during pregnancy and infants' neurodevelopment could be mediated by the maternal–fetal metabolism.

Gender difference in neurodevelopment has been reported, and compared with boys, the nervous system in girls adapts better to nutritional stress conditions (16–18). A previous study demonstrated the significant associations between maternal glycemia and poorer neurodevelopment in boys but not girls (19). Evidence on the gender-specific impact on the relationship between maternal MD during pregnancy and infants' neurodevelopment was not determined.

This study aimed to investigate the independent association of maternal MD and infant neurodevelopment in a prospective birth cohort adjusting for potential confounders. It could conceivably be hypothesized that (1) maternal MD is associated with a reduced risk of poor neurodevelopment in infants and (2) cord blood metabolic markers may mediate this association.

## Methods

### Study population

This study was a population-based birth cohort that recruited pregnant women from three centers in Hefei (Anhui Women and Child Health Care Hospital, the First People's Hospital of Hefei City, and the First Affiliated Hospital of Anhui Medical University) (20). Pregnant women aged 18 to 45 years, living in Hefei, with no communication barriers, and who had delivered a singleton live birth but without assisted reproductive technology were recruited. From March 2018 to January 2021, a total of 3,566 participants' baseline data have been finished. This information was completed by structured questionnaire through a face-to-face interview when the participants were 16–23 weeks pregnant. Participants' essential characteristics including sociodemographic characteristics, perinatal health status, and lifestyle during pregnancy were collected using questionnaires. Venous cord blood samples were collected for the detection of cord metabolic markers at delivery, and the delivery information was copied from the medical system. The childhood follow-up was conducted by community physicians at 6 months and 12 months of age, which was also completed by the questionnaire. This study was approved by the Ethics Committee of Anhui Medical University (number: 20180092). All pregnant women ascertained their willingness to provide written informed consent for themselves.

During the pregnancy follow-up period, 297 participants were excluded due to being diagnosed with liver, renal, and thyroid dysfunctions or diabetes before pregnancy, being diagnosed with liver or renal dysfunction during pregnancy, or not having complete dietary data. A total of 342 participants were excluded because of gestational age of < 32 weeks, delivery in other hospitals, or failure to provide cord blood samples. During the postpartum follow-up period, 859 participants were excluded due to birth defects (*n* = 97), unable to complete the Ages and Stages Questionnaires, Third Edition (ASQ-3) (*n* = 373), and lost to follow-up at 12 months (*n* = 389). Finally, 1,471 mother–infant pairs were analyzed (Supplementary Figure 1).

### MD scoring assessment

Food consumption frequency and serving size in the past month were collected at 16–23 gestational weeks using the food frequency questionnaire (FFQ). The frequency included never or rarely, 1–2 times a week, 3–6 times a week, and more than once per day (never or rarely = 0 times/day; 1–2 times a week = 0.2/day; 3–6 times a week = 0.6/day; more than once per day = 1/day) (20). The serving sizes were described by natural portions or standard weight/volume (20).

The score was obtained by referring to the MD score modified by Bédard et al. (7). The median weekly intake of food groups was the basis of the score. Based on 39 pre-defined food groups in our study, the relevant food groups were selected and divided into six beneficial food groups (vegetables, legumes, fruits and nuts, cereal, fish, and dairy) and one detrimental food group (meat). When consumption of the beneficial food group was above the median, mothers scored 1 point, and those below the median scored 0 points. The opposite is true for meat consumption. The MD score was calculated by summing the values of the seven food groups and it ranged from 0 to 7. We divided it into low (0-3) score and high (4-7) score groups, and the high score group represented greater adherence to a Mediterranean-style diet.

### Outcome assessment

Developmental delays in the infant at 12 months were evaluated by the ASQ-3. The prematurity age has been adjusted by a standardized ASQ-3 age calculator administered (21). Mothers were responsible for completing this questionnaire, and they were helped by trained investigators when they could not understand the questions clearly.

The ASQ-3 is validated to screen for delays in five domains (communication, gross motor, fine motor, personal–social functioning, and problem-solving ability). Each question had three choices (“yes,” “sometimes,” and “not yet”). If infants demonstrated the ability to do the specific activity described by the item, mothers could choose “Yes” and get 10 points. “Sometimes” marked that the skill was emerging and corresponded to 5 points, and “not yet” indicated that the child had not yet shown the ability to do that specific activity and scored 0 points. We calculated the sum of the scores for each domain and then defined cutoff points based on their scores (reducing the mean score by two standard deviations). Infants were thought to “fail” the screening for potential developmental delays when their scores were below the cutoff points (16). “Any fail” meant that the infants failed in at least one ASQ-3 domain.

### Cord serum metabolic markers measures

The previous collected venous cord blood samples were detected for cord serum metabolic markers, including C-peptide, high-density lipoprotein (HDL)-cholesterol, low-density lipoprotein (LDL)-cholesterol, total cholesterol (TC), and triacylglycerol (TG). The levels of serum C-peptide were tested by an immunoassay (Auto DELFIA, PerkinElmer). Cord blood HDL-cholesterol, LDL-cholesterol, TC, and TG were measured using an automatic analyzer (Beckman Coulter, Brea, CA, USA).

### Potential covariates

Potential covariates were considered characteristics that have an established or potential association with exposures or outcomes of interest, including (A) characteristics of pregnant women: mothers' age at delivery (< 30 and ≥30 years), education (≤ 12 and >12 years), household income (< 6,000 and ≥6,000 yuan), gravidity and parity history, pre-pregnancy body mass index (BMI; < 24 kg/m^2^ and ≥24 kg/m^2^), gestational diabetes (yes and no), blood pressure (systolic and diastolic), anemia during pregnancy (yes and no), depressive symptom (yes and no), the frequency of physical exercise, and the supplement of vitamin D, folic acid, and iron and (B) infant characteristics: mode of delivery (cesarean section and eutocia), birth weight, gender, delivery pregnancy week, and infant feeding status at 6 months (exclusive breastfeeding and other feedings).

### Statistical analysis

The distributions of the maternal and infant variables across the maternal MD score categories (high score vs. low score) were compared using the chi-square tests for categorical variables and *t*-tests for continuous variables, and we used the same method to compare the difference of variables between completed follow-up and lost follow-up.

The association of MD scores (categorical and continuous) with developmental delay in infants was shown based on the chi-square tests and logistic regression. Stratified logistic analyses were used to estimate the association of MD score with ASQ-3 failures according to infant gender. Stratified multiple linear regression analyses were used to estimate the association of MD score and continuous metabolic markers according to infant gender.

Cord serum C-peptide of >90th percentile was used for the diagnosis of fetal hyperinsulinemia (22, 23), which was usually used to divide continuous C-peptide into categorical variables. For the cord blood lipids, referring to correlative studies, we used the 75th percentile as the cutpoint finally (24, 25), and the percentiles were produced through all participants. Then, we used logistic regression to assess the relationship between the categorical MD score and categorical cord blood markers and the associations of categorical cord blood markers with ASQ-3 failures.

Mediation analysis was completed using the SPSS PROCESS plug-in. We evaluated the role of log-transformed C-peptide in the relationship between maternal MD score and the failure of the communication domain. In the sensitivity analysis, we conducted the association between MD score with the failure of communication domain in different pre-pregnancy BMI subgroups and GDM (yes and no) subgroups, and stratified logistic regression was used for sensitivity analysis. All analyses were performed using SPSS version 26.0 software (IBM Corp, Armonk, NY, United States).

## Results

Bivariate descriptive analysis (Table 1) revealed that mothers in the high MD score group (*n* = 578) were more highly educated, with higher household incomes and more frequent physical exercise than those in the low MD score group (*n* = 893). Meanwhile, their children scored higher in communication, fine motor, and personal social domain. When we compared the characteristics between mothers included in the study and those excluded, the result revealed that those excluded were younger and had lower education levels (Supplementary Table 1).

**Table 1:** Characteristics of mothers and infants.

**Characteristics**	**Participants**		***P*-value^#^**
	**Low score (*****n*** = **893)**^a^	**High score (*****n*** = **578)**^b^	
**Demographics**			
Age ≥ 30 years, *n* (%)	377 (42.2)	249 (43.1)	0.744
Education ≤ 12 years, *n* (%)	311(34.8)	164 (28.4)	**0.010**
Household income < 6,000 RMB/month, *n* (%)	492 (55.1)	270 (46.7	**0.002**
**Perinatal health status**			
Multipara, *n* (%)	443 (49.6)	279 (48.3)	0.616
Pre-pregnancy BMI ≥ 24 kg/m^2^, *n* (%)	148 (16.6)	109 (18.9)	0.260
Gestational diabetes, *n* (%)	179 (20.0)	128 (22.2)	0.355
Systolic blood pressure, mean ± SD, mmHg	111 ± 9.6	110 ± 10.2	0.859
Diastolic blood pressure, mean ± SD, mmHg	69 ± 7.2	69 ± 7.9	0.594
Anemia during pregnancy, *n* (%)	353 (39.5)	223 (38.6)	0.737
Depressive symptom, *n* (%)	128 (14.3)	65 (11.2)	0.087
**Pregnancy lifestyle**			
Physical activity < 3 days/week, *n* (%)	403 (45.1)	183 (31.7)	**< 0.001**
Vitamin D supplement < 1 time/week, *n* (%)	749 (83.9)	482 (83.4)	0.806
Folic acid supplement < 1 time/week, *n* (%)	70 (7.8)	43 (7.4)	0.779
Iron supplement < 1 time/week, *n* (%)	594 (66.5)	376 (65.1)	0.562
**Infant characteristics**			
Cesarean section, *n* (%)	285 (31.9)	180 (31.1)	0.517
Female, *n* (%)	422 (47.3)	255 (44.1)	0.238
Gestational week at birth, mean ± SD, week	39.3 ± 1.2	39.3 ± 1.2	0.845
Birth weight, mean ± SD, g	3,428 ± 443	3,417 ± 415	0.321
Exclusive breastfeeding at 6 months, *n* (%)	309 (34.6)	211 (36.5)	0.242
**ASQ-3 at 12 months**			
Scores for communication domain, mean ± SD	53.5 ± 9.2	55.5 ± 6.8	**< 0.001**
Scores for gross motor domain, mean ± SD	45.3 ± 13.7	45.6 ± 13.7	0.732
Scores for fine motor domain, mean ± SD	51.8 ± 9.6	53.2 ± 8.9	**0.004**
Scores for problem-solving domain, mean ± SD	50.3 ± 10.8	51.4 ± 9.7	0.053
Scores for personal social domain, mean ± SD	47.9 ± 12.5	49.3 ± 10.7	**0.029**

Data are presented as mean ± SD or n (%). ^#^Chi-squared tests were used for categorical variables, and t-tests were used for continuous variables. ^a^0–3 score. ^b^4–7 score. The bold values indicate the results were statistical significance.

### Maternal MD and ASQ failure of infants

Compared with the low score group, the high score group was less likely to be ASQ-3 failure in the communication domain (2.6 vs. 7.4%, *P* < 0.001), problem-solving domain (1.9 vs. 3.8%, *P* = 0.038), personal–social domain (2.1 vs. 5.4%, *P* = 0.002), and any failure (11.8 vs. 17.4%, *P* = 0.003) (Table 2). In unadjusted analyses (Model 1), the higher MD score was associated with lower RR for the developmental delays in infants at 12 months in the communication domain [RR with 95% CI: 0.33 (0.19, 0.59)], problem-solving domain [RR with 95% CI: 0.49 (0.25, 0.98)], personal–social domain [RR with 95% CI: 0.37 (0.20, 0.71)], and any failure [RR with 95% CI: 0.63 (0.47, 0.86)]. This association remained in the communication domain [RR with 95% CI: 0.30 (0.14, 0.64)] and personal–social domain [RR with 95% CI: 0.40 (0.17, 0.95)] after controlling for some potential confounders (Model 2). When we controlled more potential confounders, there is only the relationship between the MD score and the communication domain [RR with 95% CI: 0.34 (0.16, 0.72)] which proved to be meaningful (Model 3).

**Table 2:** Associations of Mediterranean diet score during pregnancy with developmental delays in infants at 12 months.

**ASQ-3 failure**	**Mediterranean diet score (*****n*** = **1,471)**		**Model 1^a^**	**Model 2^b^**	**Model 3^c^**
	**Low score**^d^ ***n*** **(%)**	**High score**^e^ ***n*** **(%)**	**RR (95% CI)**	**RR (95% CI)**	**RR (95% CI)**
Communication	66 (7.4)	15 (2.6)	**0.33 (0.19, 0.59)**	**0.30 (0.14, 0.64)**	**0.34 (0.16, 0.72)**
Gross motor	41 (4.6)	28 (4.8)	1.06 (0.65, 1.73)	1.34 (0.74, 2.45)	1.35 (0.73, 2.49)
Fine motor	48 (5.4)	22 (3.8)	0.70 (0.42, 1.17)	0.74 (0.38, 1.46)	0.79 (0.40, 1.53)
Problem-solving	34 (3.8)	11 (1.9)	**0.49 (0.25, 0.98)**	0.81 (0.36, 1.86)	0.87 (0.38, 2.00)
Personal social	48 (5.4)	12 (2.1)	**0.37 (0.20, 0.71)**	**0.40 (0.17, 0.95)**	0.44 (0.18, 1.04)
Any failure	155 (17.4)	68 (11.8)	**0.63 (0.47, 0.86)**	0.71 (0.49, 1.05)	0.75 (0.51, 1.11)

n (%) for the frequency of ASQ-3 failures.

Relative risks (RR) with 95% CI for the dichotomous outcomes of ASQ-3 failures are for Mediterranean diet score during pregnancy (4–7 vs. 0–3).

^a^Model 1: Unadjusted.

^b^Model 2: Adjusted confounders included maternal age, education, household income, pre-pregnancy BMI, gestational diabetes, blood pressure (systolic and diastolic), anemia during pregnancy, depressive symptoms, maternal physical exercise, the supplement of nutrients such as vitamin D, folic acid, as well as iron and pregnancy history.

^c^Model 3: Model 2, additionally adjusted for delivery mode, gender, gestational week, birth weight, and the patterns of infant feeding.

^d^0–3 score.

^e^4–7 score.

The bold values indicate the results were statistical significance.

### The role of cord serum markers in the association between maternal MD and ASQ-3 failure of infants

**Figure 1:**
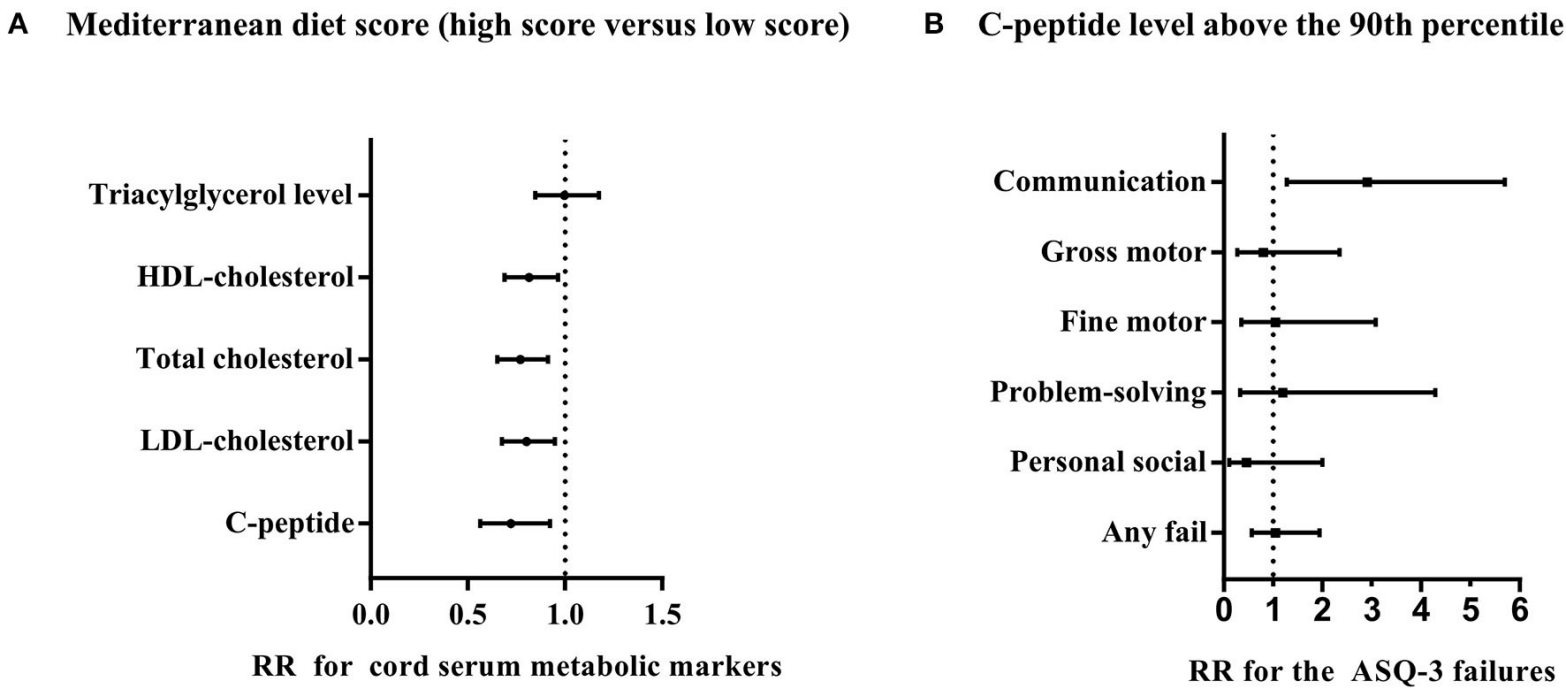
Associations of Mediterranean diet score with cord blood markers and associations of cord blood markers with ASQ-3 failures. **(A)** RRs with 95% CI for the dichotomous outcomes of cord blood level are for Mediterranean diet score (4–7 vs. 0–3). **(B)** RRs with 95% CI for the dichotomous outcomes of ASQ-3 failures including communication domain, gross motor domain, fine motor domain, problem-solving domain, personal social domain, and any failure for the cord C-peptide level above the 90th percentile. Both **(A, B)** adjusted confounders including maternal age, education, household income, pre-pregnancy BMI, gestational diabetes, blood pressure (systolic and diastolic), anemia during pregnancy, depressive symptoms, pregnancy history, maternal physical exercise, and the supplement of nutrients, such as vitamin D, folic acid, and iron, and **(B)** additionally adjusted delivery mode, gender, gestational week, and birth weight, and the patterns of infant feeding.

In addition, our study showed that the associations of MD scores with cord serum markers and associations of cord serum markers with ASQ-3 failures were meaningful (Figure 1). Mothers with low MD scores had an increased risk of higher cord serum marker levels in adjusted analyses the C-peptide level above the 90th percentile [RR with 95% CI: 0.72 (0.56, 0.92)], the LDL-cholesterol level above the 75th percentile [RR with 95% CI: 0.80 (0.68, 0.95)], the total cholesterol level above the 75th percentile [RR with 95% CI: 0.77 (0.65, 0.91)], and the HDL-cholesterol level above the 75th percentile [RR with 95% CI: 0.82 (0.67, 0.97)], except for triacylglycerol level above the 75th percentile [RR with 95% CI: 0.99 (0.85, 1.17)]) (Figure 1A). When the relationship between cord serum metabolic markers and ASQ-3 failures were analyzed, we found that only the cord serum C-peptide level above the 90th percentile and the failure of the communication domain was significative [RR with 95% CI: 2.90 (1.28, 5.69)] (Figure 1B). Furthermore, we analyzed that the influence of MD during pregnancy on log-transformed cord serum C-peptide behaves differently by gender [β with 95% CI for boys: −0.028 (−0.055, −0.001); for girls: −0.003 (−0.029, 0.024)] (Supplementary Table 2). There were no significant differences for the ASQ-3 failure to other cord serum metabolic markers (Supplementary Figure 2).

### Investigation to explore gender-specific associations

In addition, we revealed that the incidence of ASQ-3 failures differed between boys and girls. Boys' ASQ-3 failures are characteristics of high incidence than girls' in the communication domain (9.8 vs. 3.1%, *P* < 0.001), personal–social domain (6.8 vs. 2.5%, *P* = 0.006), and any failure (20.2 vs. 12.4%, *P* = 0.004). When we stratified/interacted with the analyses of the MD and ASQ-3 failures by children gender, the maternal MD was significantly associated with the communication domain only among boys [RR with 95% CI: 0.34 (0.14, 0.84)] but not among girls [RR with 95% CI: 0.26 (0.06, 1.18)] after adjusting for confounders (Table 3).

**Table 3:** Gender-specific effects on the relationship of Mediterranean diet score (4–7 vs. 0–3) with ASQ-3 failures.

**ASQ-3 failure**	**Boys (*****n*** = **794)**			**Girls (*****n*** = **677)**			***P-*value for interaction**
	***n*** **(%)**		**RR (95% CI)**	***n*** **(%)**		**RR (95% CI)**	
	**Low score** ^a^	**High score** ^b^		**Low score** ^a^	**HIGH score** ^b^		
Communication	**46 (9.8)**	**10 (3.1)**	**0.34 (0.14, 0.84)**	20 (4.7)	5 (2.0)	0.26 (0.06, 1.18)	0.109
Gross motor	19 (4.0)	12 (3.7)	1.59 (0.65, 3.93)	22 (5.2)	14 (5.5)	1.11 (0.46, 2.68)	0.543
Fine motor	30 (6.4)	13 (4.0)	0.72 (0.28, 1.83)	18 (4.3)	9 (3.5)	0.84 (0.30, 2.35)	0.481
Problem-solving	16 (3.4)	9 (2.8)	1.53 (0.50, 4.64)	18 (4.3)	4 (1.6)	0.35 (0.07, 1.76)	0.120
Personal social	**32 (6.8)**	**8 (2.5)**	0.45 (0.15, 1.30)	16 (3.8)	4 (1.6)	0.39 (0.07, 2.08)	0.322
Any fail	**95 (20.2)**	**40 (12.4)**	0.68 (0.41, 1.14)	60 (14.2)	28 (11.0)	0.80 (0.43, 1.48)	0.236

n (%) for the frequency of ASQ-3 failures.

^a^0-3 score.

^b^4-7 score.

RRs with 95% CI for the dichotomous outcomes of ASQ-3 failures are for Mediterranean diet score during pregnancy (4–7 vs. 0–3). Models are adjusted for maternal age, education, household income, pre-pregnancy BMI, gestational diabetes, blood pressure (systolic and diastolic), anemia during pregnancy, depressive symptoms, maternal physical exercise, the supplement of nutrients such as vitamin D, folic acid, as well as iron, pregnancy history, delivery mode, gestational week, birth weight, and the patterns of infants feeding. P-value for gender × Mediterranean diet score interaction term from the regression model including boys and girls.

The bold values indicate the results were statistical significance.

### Sensitivity analysis and mediation analysis

**Figure 2:**
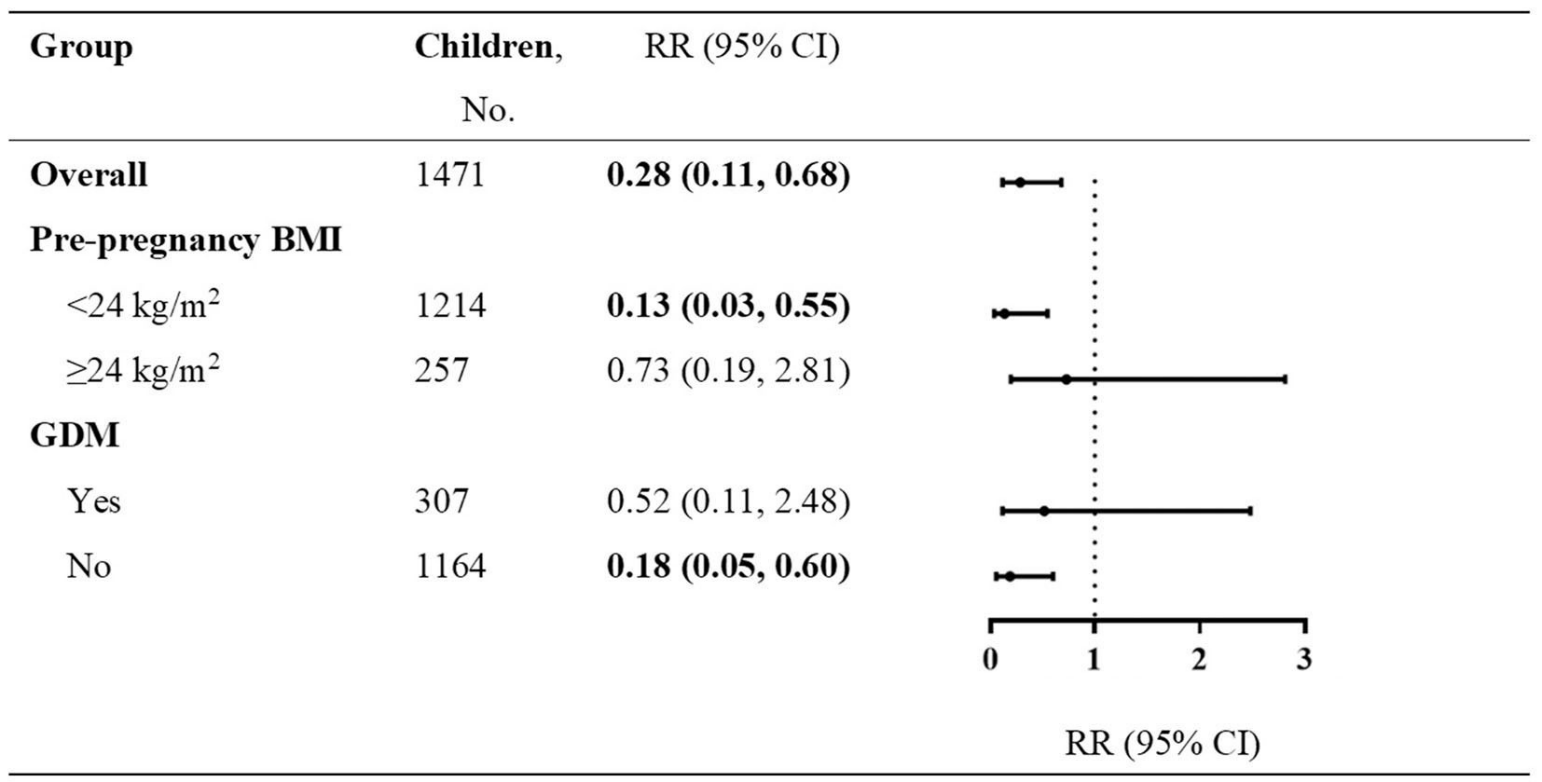
Communication domain failure in infants born to mothers with low Mediterranean diet score vs. high Mediterranean diet score. RR with 95% CI for the dichotomous outcomes of communication domain failure is for Mediterranean diet score during pregnancy (4–7 vs. 0–3). Adjusted confounders included maternal age, education, household income, blood pressure (systolic and diastolic), anemia during pregnancy, depressive symptoms, maternal physical exercise, the supplement of nutrients, such as vitamin D, folic acid, and iron, pregnancy history, delivery mode, gender, gestational week, k, birth weight, and the patterns of infant feeding.

**Figure 3:**
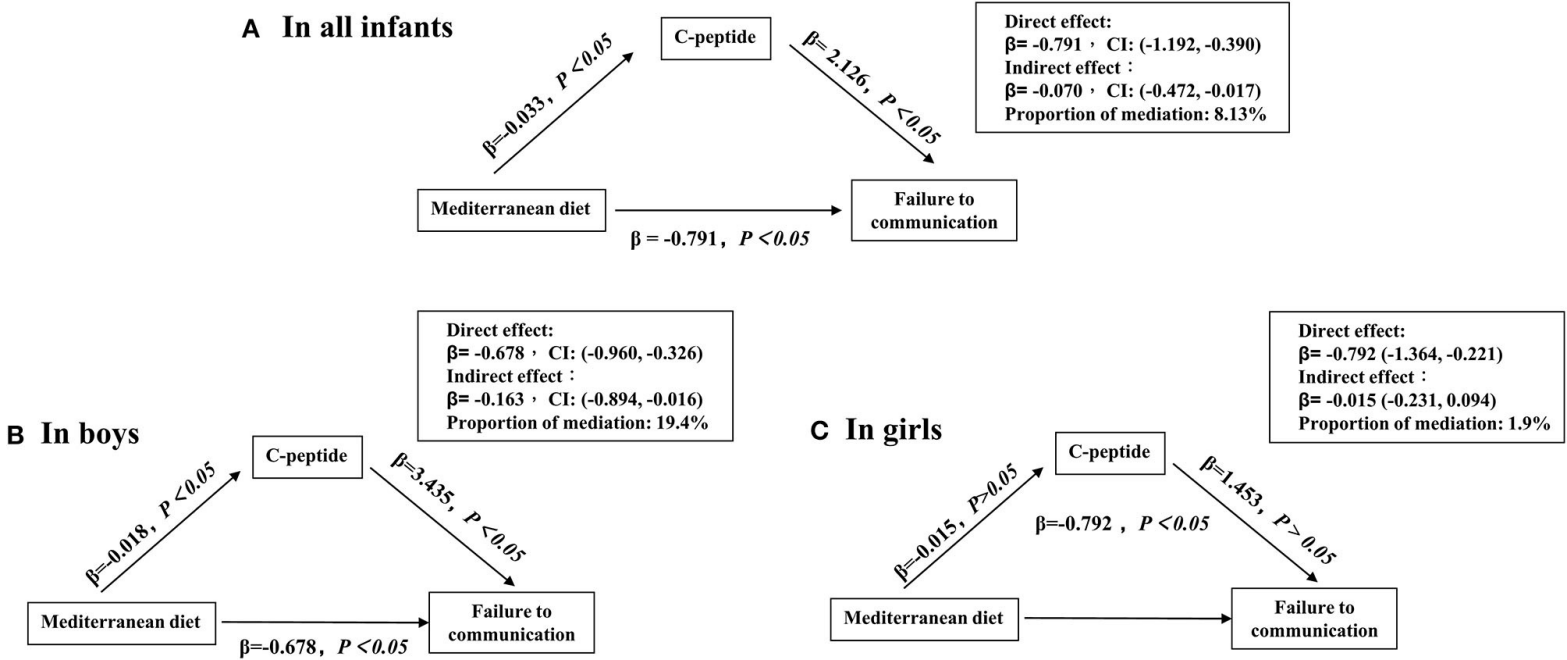
Mediation effects of cord C-peptide (loge transformed) on the relationship between maternal Mediterranean diet score and failing the communication domain. **(A–C)** Adjusted confounders included maternal age, education, household income, pre-pregnancy BMI, gestational diabetes, blood pressure (systolic and diastolic), anemia during pregnancy, depressive symptoms, maternal physical exercise, the supplement of nutrients, such as vitamin D, folic acid, and iron, pregnancy history, delivery mode, gestational week, birth weight, and patterns of infant feeding.

In sensitivity analyses, our study showed that the maternal high MD score could improve those infants' communication domain born to mothers with pre-pregnancy BMI of < 24 kg/m^2^ or without GDM (Figure 2). Finally, the mediation analysis revealed the contribution of maternal MD to the failure of the communication domain mediated by the log-transformed cord serum C-peptide [indirect effect in all infants with 95% CI: −0.070 (−0.472, −0.017); indirect effect in boys with 95% CI: −0.163 (−0.894, −0.016) and in girls with 95% CI: −0.015 (−0.231, 0.095)] (Figure 3).

## Discussion

In this prospective birth cohort study, compared with infants whose mothers have low MD scores, infants whose mothers have high MD scores had lower risks of ASQ-3 failure in the communication, problem-solving, and personal–social domain in the unadjusted model. After adjusting confounders, the association remained for the communication domain. A significant gender-specific effect in the association was observed. MD during pregnancy appears to protect neurodevelopment in boys significantly rather than in girls. Furthermore, the relationship between low MD scores and the failure of the communication domain in boys may be partly mediated by cord serum C-peptide.

As a western dietary pattern, MD was associated with reduced risks of adverse pregnancy outcomes (26), but these associations in Chinese mother–infant pairs are uncertain. Research shows that higher adherence to the MD has been relevant to better blood glucose regulation during pregnancy, lower risk of preterm birth, and lower incidence of fetal growth restriction (27). Recent evidence raised concerns about the association between dietary and personal cognitive function, mood, behavior, and neurodevelopment (28–31). The potential protective impacts of the Mediterranean diet on offspring neurodevelopment during pregnancy may be due to the consumption of beneficial foods (vegetables, legumes, fruits and nuts, cereal, fish, and dairy) or nutrients (such as omega-3 fatty acids). An RCT study found that infants of mothers who had eaten 200 g of cod two times a week for 16 weeks had a better cognitive composite compared with the control group (31). A diet rich in omega-3 fatty acids may support neurodevelopment in humans (32). Therefore, our study extended previous work for dietary guidelines during pregnancy.

Evidence presented that MD was related to maternal glucose metabolism during pregnancy (12, 13). Data exhibited that maternal metabolism may play an important role in children's neurodevelopment (23, 33, 34). However, information linking cord blood metabolism and infant neurodevelopment is lacking (16). In this context, we found that cord serum C-peptide may be the mediator in the correlation between maternal MD and failure of the communication domain. Although high cord C-peptide has been proven to contribute to some adverse outcomes (for example, poor neurodevelopmental, slower growth rate, and childhood adiposity) (23, 35, 36), our study is the first to highlight the association of metabolic regulation between maternal diet and infant neurodevelopment. Animal and epidemical studies found that the elevating levels of serum C-peptide might contribute to the abnormality of the nitric oxide signaling pathway and glucose homeostasis, which might explain the impaired brain structure and function (37–39).

Furthermore, epidemiological and animal studies showed that boys may be more sensitive to suboptimal intrauterine exposure (19). Potential gender-specific sensitivity might lead to profound and lasting differential long-term health consequences. Nonetheless, evidence on gender dimorphism relationships between diet and infant development is limited. The association between MD and infant neurodevelopment was significant in boys but not girls. While our results were partly limited to the sample size and seemed no significant difference in interaction analysis, we reported for the first time the potential gender-specific impacts on the association between maternal MD and neurodevelopmental outcomes, and our study showed that maternal MD could not affect the cord serum C-peptide levels in girls. Therefore, the gender difference in MD affecting neurodevelopment may be explained by this metabolic pathway.

### Strengths and limitations

This study has several strengths. The population-based prospective cohort study has rich information on sociodemographic characteristics, which helped reduce residual confounding. Moreover, different from a single nutrient, the use of dietary patterns captured the overall quality of the diet. Meanwhile, our study highlights that cord serum metabolites could play a mediation role in infant neurodevelopment. Furthermore, gender-specific was observed in association analysis and mediation analysis.

Several potential limitations merit discussion. First, ASQ-3 was used but lacked systematic neurodevelopmental assessments. Nonetheless, the ASQ-3 was demonstrated to identify early developmental delays and has an intimate correlation with the Bayley Scale of Infant and Toddler Development, Third Edition (Bayley-III) (40). Second, measurement errors in dietary assessments are also a concern. The FFQ relies considerably on the participants' memory and could be subject to recall errors. However, FFQ remains the most appropriate tool for assessing dietary data in epidemiological studies (41). Third, only several conventional cord metabolic markers (including C-peptide, high-density lipoprotein-cholesterol, low-density lipoprotein-cholesterol, total cholesterol, and triglycerides) were measured in this cohort study due to the limited research funding. More markers such as glucose and leptin would be added in future studies. Finally, we did not consider the influence of complementary foods, but we adjusted for the infant feeding patterns.

## Conclusion

In this prospective birth cohort study, infants born to mothers who adhere to the Mediterranean diet during pregnancy may have better neurodevelopment. We found novel evidence that the possible mechanism being greater adherence to an MD in pregnancy may mean lower cord serum C-peptide, which is associated with better neurodevelopment in infants. These results have important implications for targeting prevention in improving maternal and fetal outcomes.

## Data availability statement

The original contributions presented in the study are included in the article/Supplementary material, further inquiries can be directed to the corresponding authors.

## Ethics statement

The studies involving human participants were reviewed and approved by the Ethics Committee of Anhui Medical University (number: 20180092). The patients/participants provided their written informed consent to participate in this study.

## Author contributions

F-cD collected and analyzed data and wrote the manuscript. PW provided input in terms of statistical analysis and critically reviewed the manuscript. QL, LZ, L-jY, and LW were responsible for collecting data and contributing to clinical assessments. R-xT designed the study and assisted with the data collection. PZ designed the study, was involved in the baseline and follow-up supervision, and critically reviewed the manuscript. All authors have read and approved the final manuscript.

## References

[1] GBD 2016 Neurology Collaborators. Global, regional, and national burden of neurological disorders, 1990-2016: a systematic analysis for the Global Burden of Disease Study 2016. Lancet Neurol. (2019) 18:459–80. 10.1016/S1474-4422(18)30499-X 30879893 PMC6459001

[2] World Health Organization. Children and Neurodevelopmental Behavioural Intellectual Disorders (2021). Available online at: https://www.who.int/publications/i/item-/children-neurodevelopmental-behavioural-intellectual-disorders (accessed January 18, 2021).

[3] Forrest CB Riley AW. Childhood origins of adult health: a basis for life-course health policy. Health Aff. (2004) 23:155–64. 10.1377/hlthaff.23.5.155 15371381

[4] Fleming TP Watkins AJ Velazquez MA Mathers JC Prentice AM Stephenson J. Origins of lifetime health around the time of conception: causes and consequences. Lancet. (2018) 391:1842–52. 10.1016/S0140-6736(18)30312-X 29673874 PMC5975952

[5] Prado EL Larson LM Cox K Bettencourt K Kubes JN Shankar AH. Do effects of early life interventions on linear growth correspond to effects on neurobehavioural development? A systematic review and meta-analysis. Lancet Glob Health. (2019) 7:e1398–413. 10.1016/S2214-109X(19)30361-4 31537370

[6] Steenweg-de Graaff J Tiemeier H Steegers-Theunissen RP Hofman A Jaddoe VW Verhulst FC. Maternal dietary patterns during pregnancy and child internalising and externalising problems. The generation R Study. Clin Nutr. (2014) 33:115–21. 10.1016/j.clnu.2013.03.002 23541912

[7] Bédard A Northstone K Henderson AJ Shaheen SO. Mediterranean diet during pregnancy and childhood respiratory and atopic outcomes: birth cohort study. Eur Respir J. (2020) 55:1901215. 10.1183/13993003.01215-2019 31831586 PMC7066469

[8] Saunders L Guldner L Costet N Kadhel P Rouget F Monfort C. Effect of a Mediterranean diet during pregnancy on fetal growth and preterm delivery: results from a French Caribbean Mother-Child Cohort Study (TIMOUN). Paediatr Perinat Epidemiol. (2014) 28:235–44. 10.1111/ppe.12113 24754337

[9] Fernández-Barrés S Vrijheid M Manzano-Salgado CB Valvi D Martínez D Iñiguez C. The association of mediterranean diet during pregnancy with longitudinal body mass index trajectories and cardiometabolic risk in early childhood. J Pediatr. (2019) 206:119–27.e6. 10.1016/j.jpeds.2018.10.005 30429079

[10] Yonezawa Y Ueno F Obara T Yamashita T Ishikuro M Murakami K. Fruit and vegetable consumption before and during pregnancy and developmental delays in offspring aged 2 years in Japan. Br J Nutr. (2022) 127:1250–8. 10.1017/S0007114521002154 34121643 PMC8980726

[11] Martínez-Lapiscina EH Clavero P Toledo E Estruch R Salas-Salvadó J San Julián B. Mediterranean diet improves cognition: the PREDIMED-NAVARRA randomised trial. J Neurol Neurosurg Psychiatry. (2013) 84:1318–25. 10.1136/jnnp-2012-304792 23670794

[12] Slomski A. Mediterranean diet during pregnancy. JAMA. (2019) 322:1134. 10.1001/jama.2019.13918 31550027

[13] Guasch-Ferré M Willett WC. The Mediterranean diet and health: a comprehensive overview. J Intern Med. (2021) 290:549–66. 10.1111/joim.13333 34423871

[14] Lowe WL Jr Bain JR Nodzenski M Reisetter AC Muehlbauer MJ Stevens RD. Maternal BMI and glycemia impact the fetal metabolome. Diabetes Care. (2017) 40:902–10. 10.2337/dc16-2452 28637888 PMC5481987

[15] Lee IL Barr ELM Longmore D Barzi F Brown ADH Connors C. Cord blood metabolic markers are strong mediators of the effect of maternal adiposity on fetal growth in pregnancies across the glucose tolerance spectrum: the PANDORA study. Diabetologia. (2020) 63:497–507. 10.1007/s00125-019-05079-2 31915893

[16] Wang P Xie J Jiao XC Ma SS Liu Y Yin WJ. Maternal glycemia during pregnancy and early offspring development: a prospective birth cohort study. J Clin Endocrinol Metab. (2021) 106:2279–90. 10.1210/clinem/dgab331 33982055

[17] Xiang AH Wang X Martinez MP Walthall JC Curry ES Page K. Association of maternal diabetes with autism in offspring. JAMA. (2015) 313:1425–34. 10.1001/jama.2015.2707 25871668

[18] Valent D Yeste N Hernández-Castellano LE Arroyo L Wu W García-Contreras C. SWATH-MS quantitative proteomic investigation of intrauterine growth restriction in a porcine model reveals sex differences in hippocampus development. J Proteomics. (2019) 204:103391. 10.1016/j.jprot.2019.103391 31129268

[19] Li S Zhu Y Yeung E Chavarro JE Yuan C Field AE. Offspring risk of obesity in childhood, adolescence and adulthood in relation to gestational diabetes mellitus: a sex-specific association. Int J Epidemiol. (2017) 46:1533–41. 10.1093/ije/dyx151 29024955 PMC5837775

[20] Yin WJ Yu LJ Wu L Zhang L Li Q Dai FC. Adequate 25(OH)D moderates the relationship between dietary inflammatory potential and cardiovascular health risk during the second trimester of pregnancy. Front Nutr. (2022) 9:952652. 10.3389/fnut.2022.952652 35967812 PMC9372498

[21] Squires J Bricker D Twombly E Potter L. ASQ-3 User's Guide. Lane County, OR: Brookes Publishing (2009).

[22] Metzger BE Lowe LP Dyer AR Trimble ER Chaovarindr U Coustan DR. Hyperglycemia and adverse pregnancy outcomes. N Engl J Med. (2008) 358:1991–2002. 10.1056/NEJMoa0707943 18463375

[23] Daraki V Roumeliotaki T Koutra K Georgiou V Kampouri M Kyriklaki A. Effect of parental obesity and gestational diabetes on child neuropsychological and behavioral development at 4 years of age: the Rhea mother-child cohort, Crete, Greece. Eur Child Adolesc Psychiatry. (2017) 26:703–14. 10.1007/s00787-016-0934-2 28050707

[24] Schubert J Lindahl B Melhus H Renlund H Leosdottir M Yari A. Low-density lipoprotein cholesterol reduction and statin intensity in myocardial infarction patients and major adverse outcomes: a Swedish nationwide cohort study. Eur Heart J. (2021) 42:243–52. 10.1093/eurheartj/ehaa1011 33367526 PMC7954251

[25] Wang C Zhu W Zhu W Wei Y Su R. The associations between early pregnancy lipid profiles and pregnancy outcomes. J Perinatol. (2017) 37:127–33. 10.1038/jp.2016.191 27787507

[26] Lv S Qin R Jiang Y Lv H Lu Q Tao S. Association of maternal dietary patterns during gestation and offspring neurodevelopment. Nutrients. (2022) 14:730. 10.3390/nu14040730 35215380 PMC8878236

[27] Biagi C Nunzio MD Bordoni A Gori D Lanari M. Effect of adherence to mediterranean diet during pregnancy on children's health: a systematic review. Nutrients. (2019) 11:997. 10.3390/nu11050997 31052443 PMC6566280

[28] Hayden KM Beavers DP Steck SE Hebert JR Tabung FK Shivappa N. The association between an inflammatory diet and global cognitive function and incident dementia in older women: the women's health initiative memory study. Alzheimers Dement. (2017) 13:1187–96. 10.1016/j.jalz.2017.04.004 28531379 PMC5909961

[29] Shin JH Kim CS Cha L Kim S Lee S Chae S. Consumption of 85% cocoa dark chocolate improves mood in association with gut microbial changes in healthy adults: a randomized controlled trial. J Nutr Biochem. (2022) 99:108854. 10.1016/j.jnutbio.2021.108854 34530112

[30] Borge TC Biele G Papadopoulou E Andersen LF Jacka F Eggesbø M. The associations between maternal and child diet quality and child ADHD - findings from a large Norwegian pregnancy cohort study. BMC Psychiatry. (2021) 21:139. 10.1186/s12888-021-03130-4 33685413 PMC7941947

[31] Markhus MW Hysing M Midtbø LK Nerhus I Næss S Aakre I. Effects of two weekly servings of cod for 16 weeks in pregnancy on maternal iodine status and infant neurodevelopment: mommy's food, a randomized-controlled trial. Thyroid. (2021) 31:288–98. 10.1089/thy.2020.0115 32746774 PMC7891220

[32] Gómez-Pinilla F. Brain foods: the effects of nutrients on brain function. Nat Rev Neurosci. (2008) 9:568–78. 10.1038/nrn2421 18568016 PMC2805706

[33] Nomura Y Marks DJ Grossman B Yoon M Loudon H Stone J. Exposure to gestational diabetes mellitus and low socioeconomic status: effects on neurocognitive development and risk of attention-deficit/hyperactivity disorder in offspring. Arch Pediatr Adolesc Med. (2012) 166:337–43. 10.1001/archpediatrics.2011.784 22213602 PMC5959273

[34] Chen S Zhao S Dalman C Karlsson H Gardner R. Association of maternal diabetes with neurodevelopmental disorders: autism spectrum disorders, attention-deficit/hyperactivity disorder and intellectual disability. Int J Epidemiol. (2021) 50:459–74. 10.1093/ije/dyaa212 33221916 PMC8128461

[35] Regnault N Botton J Heude B Forhan A Hankard R Foliguet B. Higher cord C-peptide concentrations are associated with slower growth rate in the 1st year of life in girls but not in boys. Diabetes. (2011) 60:2152–9. 10.2337/db10-1189 21700880 PMC3142086

[36] Josefson JL Scholtens DM Kuang A Catalano PM Lowe LP Dyer AR. Newborn adiposity and cord blood C-peptide as mediators of the maternal metabolic environment and childhood adiposity. Diabetes Care. (2021) 44:1194–202. 10.2337/dc20-2398 33619125 PMC8132336

[37] Cotter MA Ekberg K Wahren J Cameron NE. Effects of proinsulin C-peptide in experimental diabetic neuropathy: vascular actions and modulation by nitric oxide synthase inhibition. Diabetes. (2003) 52:1812–7. 10.2337/diabetes.52.7.1812 12829651

[38] Charriaut-Marlangue C Bonnin P Pham H Loron G Leger PL Gressens P. Nitric oxide signaling in the brain: a new target for inhaled nitric oxide? Ann Neurol. (2013) 73:442–8. 10.1002/ana.23842 23495069

[39] Shah P Rahman SA Demirbilek H Güemes M Hussain K. Hyperinsulinaemic hypoglycaemia in children and adults. Lancet Diabetes Endocrinol. (2017) 5:729–42. 10.1016/S2213-8587(16)30323-0 27915035

[40] Schonhaut L Armijo I Schönstedt M Alvarez J Cordero M. Validity of the ages and stages questionnaires in term and preterm infants. Pediatrics. (2013) 131:e1468–74. 10.1542/peds.2012-3313 23629619

[41] Radwan H Hashim M Hasan H Abbas N Obaid RRS Al Ghazal H. Adherence to the Mediterranean diet during pregnancy is associated with lower odds of excessive gestational weight gain and postpartum weight retention: results of the mother-infant study cohort. Br J Nutr. (2021) 23:1–12. 10.1017/S0007114521002762 34294166

