# Supplementary material for: Mediterranean diet during pregnancy and infant neurodevelopment: A prospective birth cohort study
Source: Front Nutr. 2023 Jan 16;9:1078481. doi: 10.3389/fnut.2022.1078481 (PMC9885498; doi:10.3389/fnut.2022.1078481)
Supplement: Supplementary file 1 [file Data_Sheet_1.docx]

**Supplementary**

**Supplementary Table 1. Characteristics of mothers and infants between** **included and excluded**

| **Characteristics** | **Participants** | | *P* value^#^ |
| --- | --- | --- | --- |
|  | **Included** (n=1471) | **Excluded** (n=2095) |  |
| **Demographics** |  |  |  |
| Age ≥ 30 years, n(%) | 626 (42.6) | 674 (32.2) | **<0.001** |
| Education ≤ 12 years, n(%) | 475 (32.3) | 836 (39.9) | **<0.001** |
| Household income < 6000 RMB/month, n(%) | 762 (51.8) | 1115 (53.2) | 0.403 |
| **Perinatal health status** |  |  |  |
| Multipara, n(%) | 722 (49.1) | 970 (46.3) | 0.102 |
| Pre-pregnancy BMI ≥ 24 kg/m^2^, n(%) | 257 (17.5) | 357 (17.0) | 0.742 |
| Gestational diabetes, n(%) | 307 (20.9) | 387 (18.5) | 0.096 |
| Systolic blood pressure, mean ± SD, mmHg | 110 ± 9.9 | 110 ± 9.9 | 0.577 |
| Diastolic blood pressure, mean ± SD, mmHg | 69 ± 7.5 | 69 ± 7.3 | 0.269 |
| Anemia during pregnancy, n(%) | 576 (39.2) | 823 (39.3) | 0.913 |
| Depressive symptom, n(%) | 178 (12.1) | 286 (13.7) | 0.175 |
| **Pregnancy lifestyle** |  |  |  |
| Physical activity < 3 days / week, n(%) | 586 (39.8) | 889 (42.4) | 0.121 |
| Vitamin D supplement < 1 time / week, n(%) | 1231 (83.7) | 1850 (88.3) | **<0.001** |
| Folic acid supplement < 1 time / week, n(%) | 113 (7.7) | 151 (7.2) | 0.594 |
| Iron supplement < 1 time / week, n(%) | 970 (65.9) | 1322 (63.1) | 0.082 |
| **Mediterranean diet score ^*^** |  |  |  |
| low scores group, n(%) | 893 (60.7) | 1245 (63.5) | 0.092 |

Data are presented as mean ± SD or n (%). ^#^: Chi-squared tests were used for categorical variables, and t-tests were used for continuous variables. **^*:^** n=3431.

**Supplementary Table 2. Associations of Mediterranean diet** **score during pregnancy with** **cord serum C-peptide**

|  | β (95% CI) | |
| --- | --- | --- |
|  | **Model 1 ^a^** | **Model 2 ^b^** |
| **Boys** | -0.018 (-0.043, 0.007) | **-0.028 (-0.055, -0.001)** |
| **Girls** | 0.001 (-0.024, 0.026) | -0.003 (-0.029, 0.024) |
| **All** | **-0.009 (-0.017, -0.002)** | **-0.009 (-0.018, -0.001)** |

β (95% CI) for cord serum C-peptide (loge transformed) are for Mediterranean diet score during pregnancy.

^a^ Model 1: Unadjusted.

^b^ Model 2: Adjusted confounders included maternal age, education, household income, pre-pregnancy BMI, gestational diabetes, blood pressure (systolic and diastolic), anemia during pregnancy, depressive symptom, maternal physical exercise and the supplement of nutrients such as vitamin D, folic acid as well as iron.


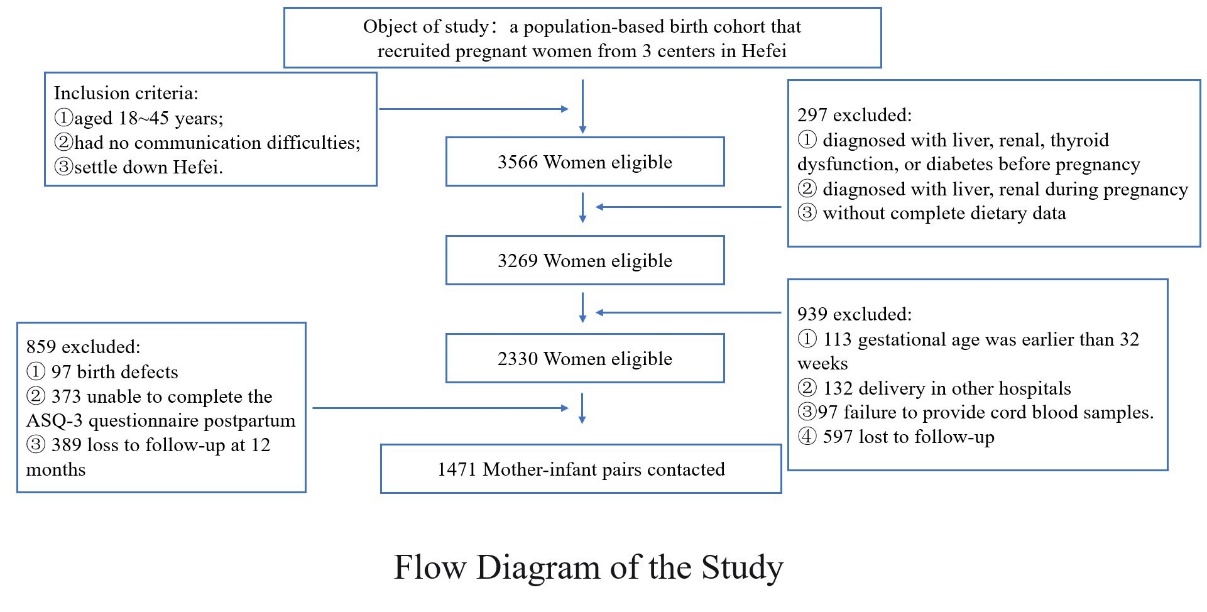


**Supplementary Figure 1:** Flow Diagram of the Study.


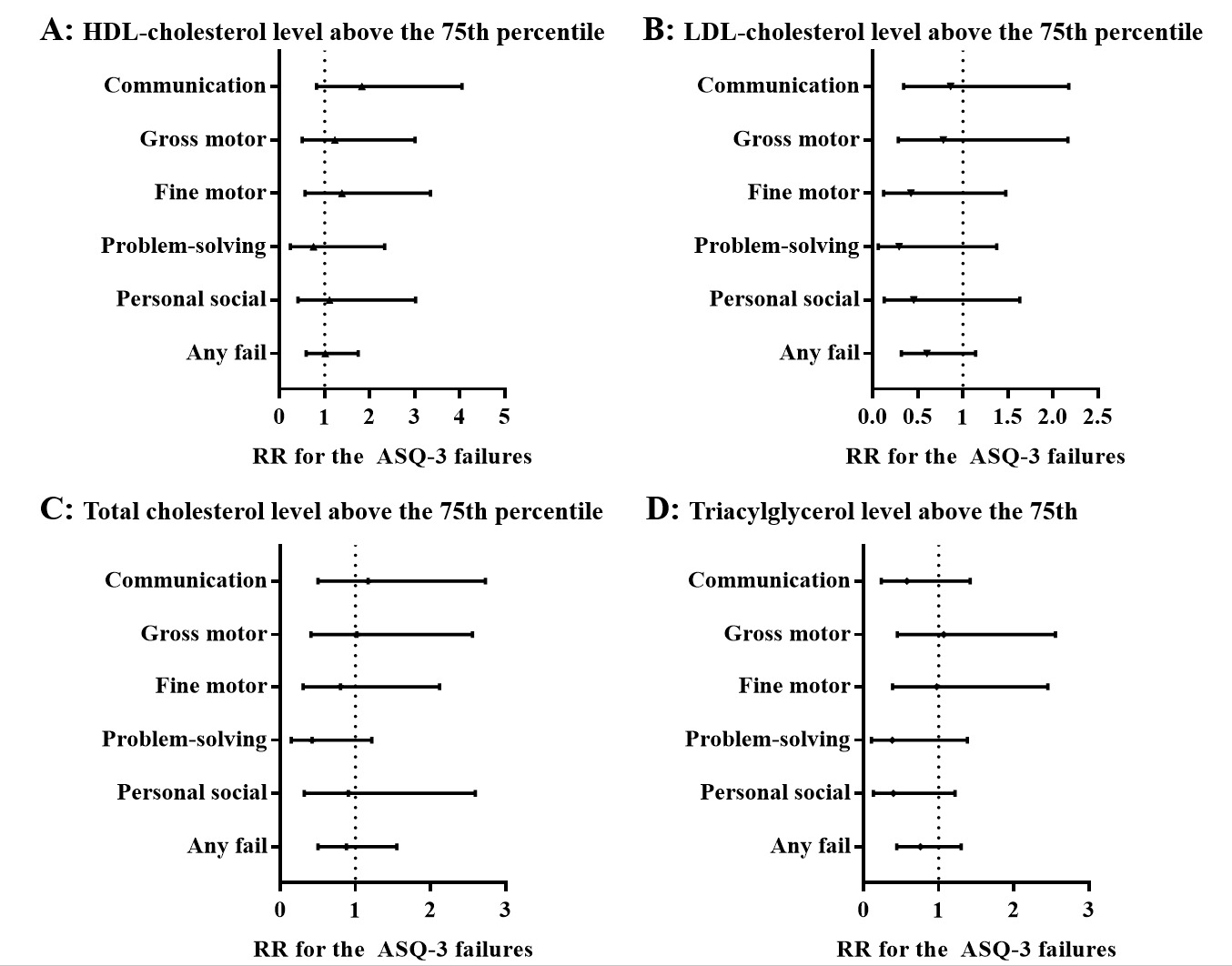


**Supplementary Figure 2. Associations of cord serum markers with ASQ failures.** (A-D) RRs with 95% CI for the dichotomous outcomes of ASQ failures including communication domain, gross motor domain, fine motor domain, problem-solving domain, personal social domain, and any fail are for cord metabolic markers (categorical), HDL-cholesterol, LDL-cholesterol, Total cholesterol, Triacylglycerol level. (A–D) adjusted confounders including maternal age, education, household income, pre-pregnancy BMI, gestational diabetes, blood pressure (systolic and diastolic), anemia during pregnancy, depressive symptom, pregnancy history, maternal physical exercise and the supplement of nutrients such as vitamin D, folic acid as well as iron.
